# Omeprazole Increases the Efficacy of Acyclovir Against Herpes Simplex Virus Type 1 and 2

**DOI:** 10.3389/fmicb.2019.02790

**Published:** 2019-12-03

**Authors:** Martin Michaelis, Malte C. Kleinschmidt, Denisa Bojkova, Holger F. Rabenau, Mark N. Wass, Jindrich Cinatl Jr.

**Affiliations:** ^1^Industrial Biotechnology Centre, School of Biosciences, University of Kent, Canterbury, United Kingdom; ^2^Institut für Medizinische Virologie, Klinikum der Goethe-Universität, Frankfurt am Main, Germany

**Keywords:** HSV, HSV-1, HSV-2, antiviral therapy, antiviral drugs, ribavirin, proton pump inhibitors

## Abstract

Omeprazole was shown to improve the anti-cancer effects of the nucleoside analogue 5-fluorouracil. Here, we combined omeprazole with the antiviral nucleoside analogues ribavirin and acyclovir. Omeprazole did not affect the antiviral effects of ribavirin in non-toxic concentrations up to 80 μg/mL but increased the acyclovir-mediated effects on herpes simplex virus 1 and 2 (HSV-1 and -2) replication in a dose-dependent manner. Omeprazole alone reduced HSV-1 and -2 titers [but not HSV-induced formation of cytopathogenic effects (CPE)] at concentrations ≥40 μg/mL. However, it exerted substantially stronger effects on acyclovir activity and also increased acyclovir activity at lower concentrations that did not directly interfere with HSV replication. Omeprazole 80 μg/mL caused a 10.8-fold (Vero cells) and 47.7-fold (HaCaT cells) decrease of the acyclovir concentrations that reduced HSV-1-induced CPE formation by 50% (IC_50_). In HSV-2-infected cells, omeprazole 80 μg/mL reduced the acyclovir IC_50_ by 7.3- (Vero cells) and 12.9-fold (HaCaT cells). In HaCaT cells, omeprazole 80 μg/mL reduced the HSV-1 titer in the presence of acyclovir 1 μg/mL by 1.6 × 10^5^-fold and the HSV-2 titer in the presence of acyclovir 2 μg/mL by 9.2 × 10^3^-fold. The proton pump inhibitors pantoprazole, rabeprazole, lansoprazole, and dexlansoprazole increased the antiviral effects of acyclovir in a similar fashion as omeprazole, indicating this to be a drug class effect. In conclusion, proton pump inhibitors increase the anti-HSV activity of acyclovir and are candidates for antiviral therapies in combination with acyclovir, in particular for topical preparations for the treatment of immunocompromised individuals who are more likely to suffer from severe complications.

## Introduction

Omeprazole and other proton pump inhibitors have been found to increase the activity of anti-cancer drugs including the nucleoside analogue 5-fluorouracil (Luciani et al., 2004; Ikemura et al., 2017). Proton pump inhibitors are the most frequently prescribed drugs for the treatment and prophylaxis of gastroesophageal reflux as well as of gastric and duodenal ulcers that are associated with hyper-acidic states. Since they are known to be well-tolerated, they were suggested as repositioning candidates for the use as part of anti-cancer therapies (Luciani et al., 2004; Ikemura et al., 2017).

Nucleoside analogues are also widely used as antiviral drugs (Chaudhuri et al., 2018; Chemaly et al., 2019). Here, we investigated the effects of omeprazole on the efficacy of the antiviral nucleoside analogues acyclovir and ribavirin. The guanosine analogue acyclovir and its pro-drug valacyclovir are used for the treatment of disease caused by herpes simplex virus 1 (HSV-1) and 2 (HSV-2) and varicella zoster virus (VZV) (Zarrouk et al., 2017). Acyclovir is activated by the viral thymidine kinase and then di- and tri-phosphorylated by cellular kinases. The active tri-phosphorylated forms of acyclovir and then specifically interferes with the viral DNA polymerase and causes chain termination (Piret and Boivin, 2016; Chen et al., 2017; Zarrouk et al., 2017). Ribavirin is a guanosine analogue that has been shown to exert broad-spectrum activity against RNA and DNA viruses including influenza viruses and West Nile virus. The mechanisms by which ribavirin interferes with virus replication are not clear and may be virus-dependent (Sidwell et al., 1972; Day et al., 2005; Beaucourt and Vignuzzi, 2014; Koh and Liang, 2014; Galli et al., 2018; Jordan et al., 2018; Keppeke et al., 2019). Our findings show that omeprazole (and other proton pump inhibitors) increase the antiviral activity of acyclovir but not that of ribavirin.

## Materials and Methods

### Cell Culture

Vero and MDCK cells were obtained from the American Type Culture Collection (ATCC, Rockville, MD, United States) and cultured at 37°C in minimum essential medium (MEM) supplemented with 10% fetal bovine serum. HaCaT cells were purchased from CLS Cell Line Services GmbH (Eppelheim, Germany) and cultivated in Iscove’s modified Dulbecco’s medium (IMDM) supplemented with 10% fetal bovine serum.

### Viruses

HSV-1 strain McIntyre and HSV-2 strain MS were both obtained from ATCC. West Nile virus (WNV) strain NY385-99 was kindly provided by Dr. J. ter Meulen (Institut für Virologie, Philipps-Universität, Marburg, Germany). Virus stocks were prepared in Vero cells grown in MEM with 4% fetal bovine serum. The influenza virus strain Influenza A/New Caledonia/20/99 (H1N1) was received from the WHO Influenza Centre (National Institute for Medical Research, London, United Kingdom). Virus stocks were prepared in MDCK cells grown in 4% fetal bovine serum. Infectious virus titers were determined by titration on MDCK cell monolayers in 96-well plates and expressed as 50% tissue culture infectious dose (TCID_50_) by the method of Spearman and Kärber (Spearman, 1908; Kärber, 1931).

### Drugs

Acyclovir was received from GlaxoSmithKline (Munich, Germany), omeprazole from AstraZeneca (Wedel, Germany), ribavirin from Valeant Pharmaceuticals Germany GmbH (Eschborn, Germany), and pantoprazole, rabeprazole, lansoprazole, and dexlansoprazole from Selleck Chemicals (via Absource Diagnostics GmbH, Munich, Germany).

### Cytopathogenic Effect (CPE) Reduction Assay

For the investigation of HSV-1- and HSV-2-induced cytopathogenic effects (CPEs), confluent Vero or HaCaT cell monolayer in 96-well microtiter plates were inoculated with HSV-1 or HSV-2 at MOI 1 or 0.1, respectively. Following a 1 h incubation period, the inoculum was removed and the drugs, either alone or in combination, were added. The virus-induced CPE was recorded microscopically after 48 h post infection.

For the investigation of WNV-induced CPEs, Vero cell monolayers were infected with MOI 0.1. Following a 1 h virus incubation period, the medium was removed and replaced by medium containing different drug concentrations. The CPE was recorded at 48 h post infection.

Confluent MDCK cell monolayers were infected with Influenza H1N1 (MOI 0.01). Following a 1 h virus incubation period, the medium was removed and infected cells were incubated in medium containing different concentrations of drugs at the respective concentration. The CPE was recorded at 24 h post infection.

Cytopathogenic effects were scored by two independent examiners and expressed in% of the untreated virus control that was defined to be 100%.

### Immunostaining

Intracellular HSV protein was evaluated by immunostaining. Cells were fixed with 60/40 ice cold methanol/acetone for 15 min. Staining was performed using a rabbit polyclonal antibody directed against HSV-1 (ab9533) and a sheep polyclonal antibody directed against HSV-2 (ab21112) in combination with biotin-conjugated secondary goat anti-rabbit (ab6720) and rabbit anti-sheep (ab6746) antibodies (all antibodies derived from Abcam, Cambridge, United Kingdom). Protein was visualized using streptavidin peroxidase complex with AEC as a substrate.

### Viability Assay

The cellular viability was assessed on confluent cell layers with the 3-(4,5-dimethyl-2-thiazolyl)-2,5-diphenyl-2H-tetrazolium bromide (MTT) assay method as described previously (Michaelis et al., 2007). The viability was expressed as percentage of non-treated control.

### Western Blot

Cells were lysed using Triton-X-100 sample buffer, and proteins were separated by SDS-PAGE. Detection occurred by using specific antibodies against HSV-1/2 gB (10B7) (sc-56987, Santa Cruz Biotechnology, Dallas, TX, United States) and α-tubulin (ab4074, Abcam, Cambridge, United Kingdom). Proteins were visualized by enhanced chemiluminescence using a commercially available kit (Bio-Rad, Feldkirchen, Germany).

### Statistics

Results are expressed as mean ± S.D. of at least three experiments. Comparisons between two groups were performed using Student’s *t*-test. Three and more groups were compared by ANOVA followed by the Student-Newman–Keuls test. *P*-values lower than 0.05 were considered to be significant.

## Results

### Effects of Omeprazole on Cell Viability

The effects of omeprazole on the viability of the investigated cell lines was tested in concentrations of up to 160 μg/mL. Omeprazole concentrations of 80 μg/mL and lower did not affect the viability of any of the tested cell lines. Omeprazole 160 μg/mL resulted in a reduction of cell viability, but the concentration that reduces cell viability by 50% (CC_50_) was not reached (Supplementary Figure S1).

### Effects of Omeprazole in Combination With Ribavirin on Cytopathogenic Effect (CPE) Formation in WNV- or Influenza A H1N1-Infected Cells

**FIGURE 1:**
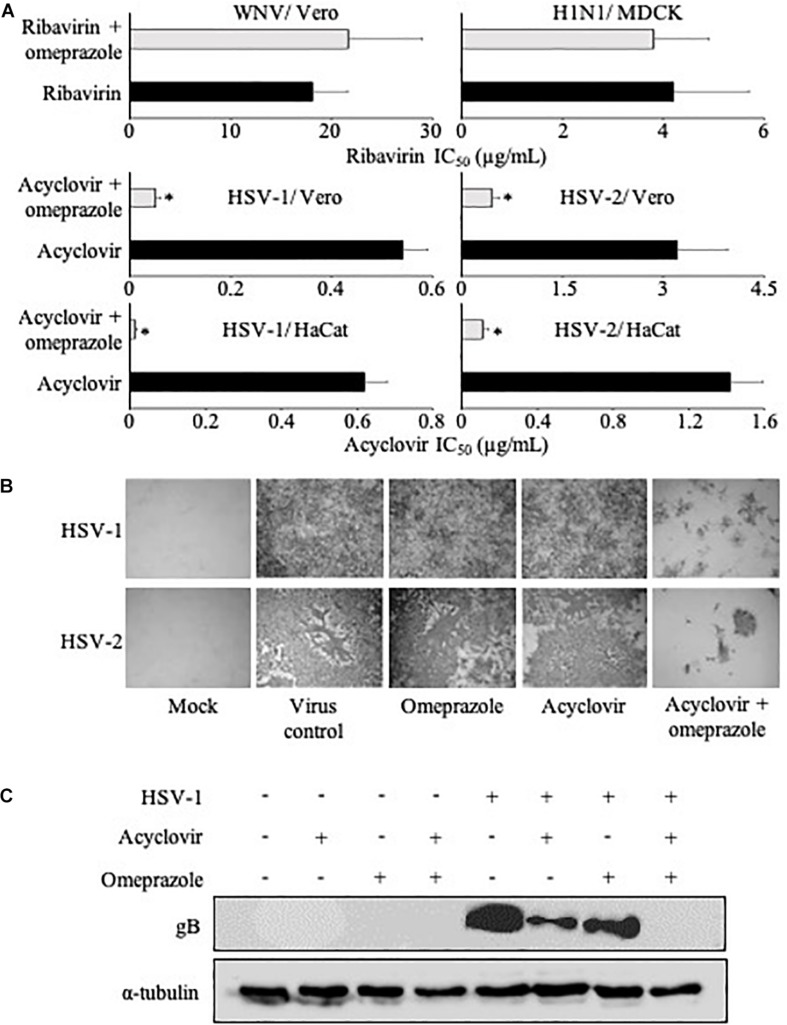
Cytopathogenic effect (CPE) formation and viral gene expression in the presence of antiviral nucleoside analogues and omeprazole. **(A)** Effects of omeprazole (80 μg/mL) on the concentrations of antiviral nucleoside analogues that reduce CPE formation by 50% (IC_50_) using West Nile virus (WNV)-infected Vero cells, influenza A H1N1-infected MDCK cells, and HSV-1- or HSV-2-infected Vero or HaCaT cells. Omeprazole alone did not reduce CPE formation. Numerical values are presented in Supplementary Table S1. **(B)** Effects of omeprazole and acyclovir on the expression of virus proteins in HSV-1- and HSV-2-infected Vero cells. HSV-1-infected cells were treated with omeprazole 80 μg/mL and/or acyclovir 0.31 μg/mL. HSV-2-infected cells were treated with omeprazole 40 μg/mL and/or acyclovir 0.6 μg/mL. **(C)** HSV gB levels in HSV-1-infected Vero cells treated with omeprazole 80 μg/mL and/or acyclovir 0.31 μg/mL as determined by Western blot 24 h post infection.

Omeprazole 80 μg/mL did not alter the effects of ribavirin on CPE formation in WNV-infected Vero cells or H1N1-infected MDCK cells (Figure 1A and Supplementary Table S1).

### Effects of Omeprazole in Combination With Acyclovir on HSV-1 and HSV-2 Replication

**FIGURE 2:**
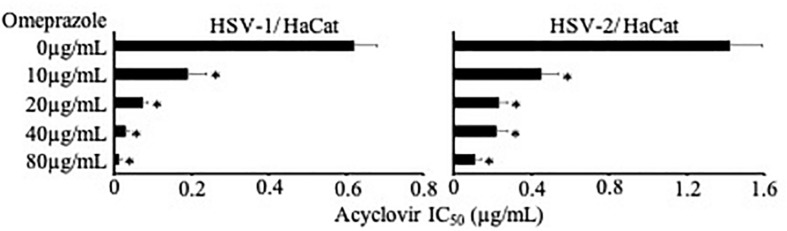
Concentration-dependent effects of omeprazole on the acyclovir IC_50_ in HSV-1- or HSV-2-infected HaCaT cells as determined by cytopathogenic effect (CPE) formation. Numerical values are presented in Supplementary Table S2. The investigated drug concentrations did not affect cell viability, neither alone or in combination. ^∗^*P* < 0.05 relative to nucleoside analogue alone.

HSV-1- and HSV-2-induced CPE formation were investigated in Vero and HaCaT cells. Vero is a continuous cell line derived from kidney epithelial cells of an African green monkey (Yasumura and Kawakita, 1963). Vero cells are interferon-deficient and used to cultivate many different viruses (Desmyter et al., 1968; Montagnon and Vincent-Falquet, 1998; Barrett et al., 2009). HaCaT is a spontaneously immortalized human keratinocyte cell line (Boukamp et al., 1988). Omeprazole on its own did not affect HSV-1- and HSV-2-induced CPE formation (Supplementary Table S1). In the presence of omeprazole 80 μg/mL, however, acyclovir concentrations that reduced CPE formation by 50% (IC_50_) were reduced by 11-fold in HSV-1-infected Vero cells and by 7-fold in HSV-2-infected Vero cells (Figure 1A and Supplementary Table S1). Further, omeprazole 80 μg/mL reduced the acyclovir IC_50_s by 48-fold in HSV-1-infected HaCaT cells and by 13-fold in HSV-2-infected HaCaT cells (Figure 1A and Supplementary Table S1). Immune staining also indicated reduced numbers of virus-infected cells after treatment with a combination of omeprazole and acyclovir compared to either single treatment (Figure 1B). In agreement, Western blot analysis demonstrated strongly reduced HSV gB protein levels in cells treated with this combination (Figure 1C). Further experiments indicated that omeprazole reduced acyclovir IC_50_s in HSV-1- and HSV-2-infected HaCaT cells in a dose-dependent fashion (Figure 2 and Supplementary Table S2).

**FIGURE 3:**
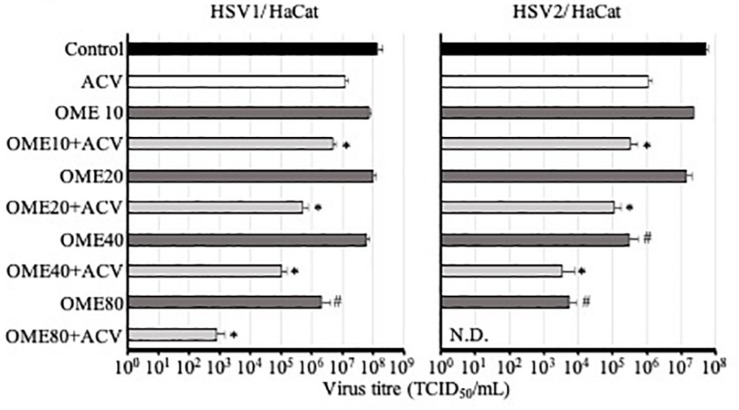
Effect of acyclovir 1 μg/mL (HSV-1) or 2 μg/mL (HSV-2) alone or in combination with varying omeprazole (OME) concentrations (μg/mL) on HSV-1 and HSV-2 titres in HaCaT cells. Numerical values are presented in Supplementary Table S3. ^∗^*P* < 0.05 relative to acyclovir alone, ^#^*P* < 0.05 relative to untreated virus control; N.D. = no detectable virus titre.

Although omeprazole did not affect the HSV-1 and HSV-2 CPEs in concentrations of up to 80 μg/mL, the determination of virus titers in Vero cells showed that 80 μg/mL omeprazole inhibited the production of infectious HSV-1 particles and that 40 and 80 μg/mL omeprazole inhibited the production of infectious HSV-2 particles. In agreement with the findings from the CPE assays, omeprazole also strongly increased the anti-HSV-1 and anti-HSV-2 effects of acyclovir. Notably, this omeprazole-induced increase of acyclovir activity was also seen at lower omeprazole concentrations, which did not directly reduce virus titers (Figure 3 and Supplementary Table S3). The investigated omeprazole and acyclovir concentrations did not affect cell viability, neither alone not in combination.

### Effects of Various Proton Pump Inhibitors on HSV-1-Induced Cytopathogenic Effects (CPEs)

**FIGURE 4:**
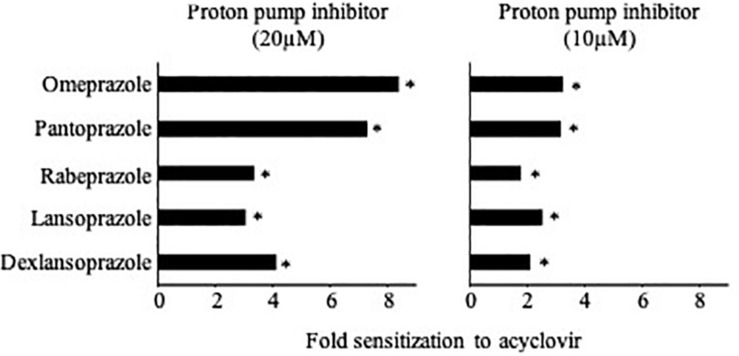
Effects of different proton pump inhibitors on acyclovir activity in HSV-1-infected HaCaT cells as indicated by cytopathogenic effect (CPE) formation. Proton pump inhibitors alone did not reduce CPE formation. Numerical values are presented in Supplementary Table S4. ^∗^*P* < 0.05 relative to acyclovir alone.

Finally, we tested the effects of the additional proton pump inhibitors pantoprazole, rabeprazole, lansoprazole, and dexlansoprazole (Li et al., 2017) on CPE formation in HSV-1-infected HaCaT cells. All tested proton pump inhibitors increased the activity of acyclovir (Figure 4 and Supplementary Table S4), which suggests that this is a drug class effect.

## Discussion

Based on previous investigations that showed that omeprazole increases the anti-cancer activity of the nucleoside analog 5-fluorouracil (Luciani et al., 2004), we here investigated the effects of omeprazole on the antiviral effects of ribavirin and acyclovir. Omeprazole did not modify ribavirin-mediated effects in H1N1 influenza A virus-infected or West Nile virus-infected cell cultures but increased the efficacy of acyclovir, a first line drug for HSV-1, HSV-2, and varicella zoster virus infection (Piret and Boivin, 2016; Klysik et al., 2018), in a dose-dependent fashion in Vero and HaCaT cells. It remains unclear why omeprazole increases the activity of acyclovir but not that of ribavirin. Differences between the compounds acyclovir and ribavirin including their mechanisms of action and/or differences between the investigated viruses may be responsible for this.

The mechanism by which omeprazole enhances the activity of acyclovir seems to differ from the mechanism by which omeprazole increases 5-fluorouracil efficacy, which was shown to be the consequence of an increase of the lysosomal pH (Luciani et al., 2004). Lysosomotropic drugs such as chloroquine and ammonium chloride are known to interfere with the infection of viruses including HSV. These drugs increase intracellular pH presumably resulting in inhibition of viral packing and maturation through *trans*-Golgi network, although their exact mechanisms of antiviral activity remain unclear (Koyama and Uchida, 1984, 1989; Johnson and Baines, 2011; Al-Bari, 2017; Salata et al., 2017). In agreement, omeprazole concentrations ≥40 μg/mL reduced HSV-1 and HSV-2 titers. However, the effects of omeprazole on the anti-HSV activity of acyclovir were more pronounced than the direct antiviral effects and lower omeprazole concentrations, which did not affect HSV-1 and HSV-2 replication, still substantially enhanced the efficacy of acyclovir. This indicates that the induction of increased acyclovir activity is not a direct consequence of antiviral activity exerted by omeprazole and may be caused by a different mechanism. Moreover, omeprazole pre-treatment was necessary to increase 5-fluorouracil activity (Luciani et al., 2004), but omeprazole and acyclovir exerted their combined activity when added at the same time 1 h post infection. This indicates that the mechanisms by which omeprazole increases 5-fluorouracil and acyclovir activity differ and that omeprazole increases the antiviral activity of acyclovir during the viral replication cycle after infection and virus internalization. The proton pump inhibitors pantoprazole, rabeprazole, lansoprazole, and dexlansoprazole increased acyclovir activity in a similar manner as omeprazole. Hence, the capacity to increase the antiviral activity of acyclovir seems to be a drug class effect, which is common to proton pump inhibitors in general. Notably, proton pump inhibitors have been shown to exert anti-inflammatory and antioxidative effects independently of their effects on H^+^/K^+^ ATPase activity, which may contribute to the increased acyclovir activity mediated by proton pump inhibitors (Balza et al., 2016; Nelson et al., 2017; Ghebre, 2018). In addition, omeprazole may inhibit DNA damage repair (Martelli et al., 1998), which may increase the efficacy of acyclovir. Therefore, proton pump inhibitors may increase acyclovir activity by mechanisms that do not involve the modulation of the lysosomal pH.

Since omeprazole is a clinically well-established drug with a preferable safety profile, it is an excellent candidate for drug repositioning strategies (Ikemura et al., 2017), and there is a need for improved therapies for HSV-1- and HSV-2-associated disease. After primary infection, HSV-1 and HSV-2 establish life-long persistence which may result in recurrent disease which typically manifests as herpes labialis or herpes genitalis and which may be associated with significant morbidity (Gnann and Whitley, 2016; Heslop et al., 2016; Klysik et al., 2018). Even in the case of herpes labialis, which is not commonly associated with severe complications, treatment success is not always satisfactory as highlighted by the introduction of topical acyclovir/hydrocortisone combinations (Nguyen et al., 2014). Omeprazole may not exert general immunosuppressive effects in the same way as hydrocortisone but to more specifically increase acyclovir activity. In immunodeficiency individuals, HSV-1 and -2 infections are often associated with more severe disease, and resistance formation to acyclovir is a severe problem (Piret and Boivin, 2016; Karrasch et al., 2018). Moreover, ocular HSV infection is a major cause of blindness in industrialized countries (Klysik et al., 2018). Thus, more effective treatment options for HSV-1- and HSV-2-caused disease are highly desirable. In this context, proton pump inhibitors are promising candidates for combination with acyclovir or valacyclovir in topical preparations. Further research will have to show to which extent effective proton pump inhibitor concentrations can also be achieved systemically. For omeprazole, maximum plasma levels have been described to reach about 8 μg/mL, when it is used for inhibition of acid secretion in the stomach (Shin and Kim, 2013). Hence, the achievement of therapeutically effective plasma concentrations seems possible, given that a dose increase may be feasible in a severe acute disease setting.

In conclusion, omeprazole and other proton pump inhibitors substantially enhance the antiviral effects of acyclovir in HSV-1- and HSV-2-infected cells. With their known safety profiles, proton pump inhibitors are promising candidates for drug repurposing approaches (Ikemura et al., 2017), in particular for topical preparations.

## Data Availability Statement

All datasets for this study are included in the article/Supplementary Material.

## Author Contributions

MM and JC designed and conducted the study. MK, DB, and JC performed experiments. All authors analyzed and curated data. MM and JC wrote the initial manuscript draft. All authors read, revised, and approved the final version of the manuscript.

## Conflict of Interest

The authors declare that the research was conducted in the absence of any commercial or financial relationships that could be construed as a potential conflict of interest.
