# Supplementary material for: Omeprazole Increases the Efficacy of Acyclovir Against Herpes Simplex Virus Type 1 and 2
Source: Front Microbiol. 2019 Dec 3;10:2790. doi: 10.3389/fmicb.2019.02790 (PMC6901432; doi:10.3389/fmicb.2019.02790)
Supplement: Supplementary file 1 [file Data_Sheet_1.PDF]

# Suppl. Figure S1

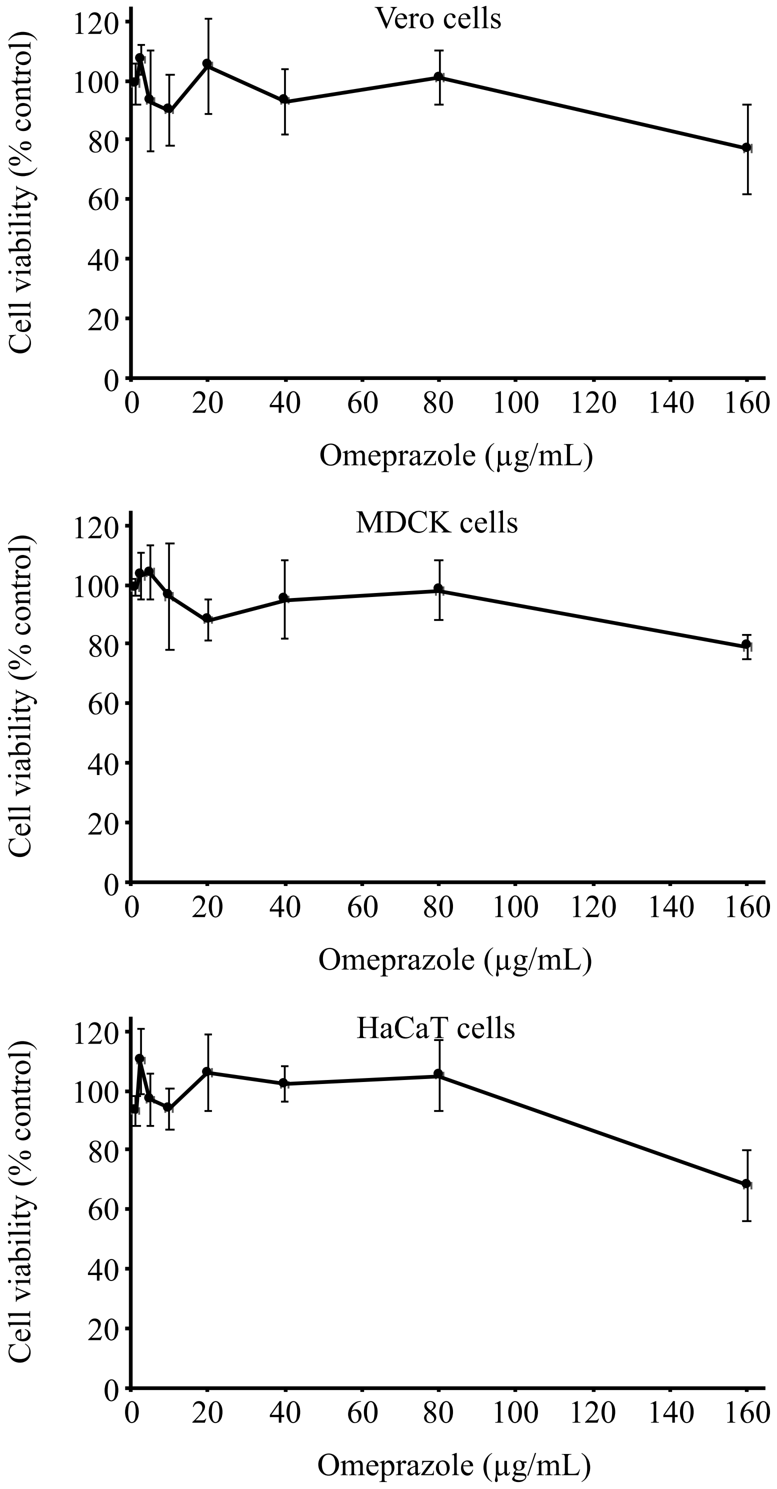


**Suppl. Figure S1**. Effects of omeprazole on cell viability as determined after 120h of incubation by MTT assay.
