# Supplementary material for: Omeprazole Increases the Efficacy of Acyclovir Against Herpes Simplex Virus Type 1 and 2
Source: Front Microbiol. 2019 Dec 3;10:2790. doi: 10.3389/fmicb.2019.02790 (PMC6901432; doi:10.3389/fmicb.2019.02790)
Supplement: Supplementary file 2 [file Data_Sheet_2.PDF]

Suppl. Table S1. Effects of omeprazole (80µg/mL) on the effects of antiviral nucleoside analogues on virus-induced cytopathogenic effect (CPE) formation. The investigated drug concentrations did not affect cell viability, neither alone, nor in combination.

A) Effects of omeprazole (80µg/mL) on the effects of ribavirin on CPE formation in West Nile virus-infected Vero cells and H1N1-infected MDCK cells.

		+ Omeprazole (80µg/mL)		
	Ribavirin (µg/mL)		Ribavirin (µg/mL)	
	IC_{50}[1]	CPE formation[2]	IC_{50}[1]	Fold change[3]
West Nile virus	18.1 ± 3.5	96 ± 18	21.6 ± 7.3	0.84
H1N1	4.2 ± 1.5	98 ± 13	3.8 ± 1.1	1.11

B) Effects of omeprazole (80µg/mL) on the effects of acyclovir on CPE formation in herpes simplex type 1 (HSV-1)- or HSV-2-infected Vero cells.

		+ Omeprazole (80µg/mL)		
	Acyclovir (µg/mL)		Acyclovir (µg/mL)	
	IC_{50}[1]	CPE formation[2]	IC_{50}[1]	Fold change[3]
HSV-1	0.62 ± 0.06	99 ± 17	0.05 ± 0.01	10.8
HSV-2	1.42 ± 0.17	99 ± 17	0.44 ± 0.12	7.3

C) Effects of omeprazole (80µg/mL) on the effects of acyclovir on CPE formation in HSV-1- or HSV-2-infected HaCaT cells.

		+ Omeprazole (80µg/mL)		
	Acyclovir (µg/mL)		Acyclovir (µg/mL)	
	IC_{50}[1]	CPE formation[2]	IC_{50}[1]	Fold change[3]
HSV-1	0.54 ± 0.05	105 ± 21	0.013 ± 0.006	47.7
HSV-2	3.20 ± 0.75	105 ± 21	0.11 ± 0.03	12.9

[1] Concentration that reduces CPE formation by 50% presented as mean ± S.D.
[2] CPE formation in the presence of omeprazole (80µg/mL) alone
[3] Fold change: IC_{50} ribavirin/ IC_{50} in the presence of omeprazole (80µg/mL)
