# Supplementary material for: Omeprazole Increases the Efficacy of Acyclovir Against Herpes Simplex Virus Type 1 and 2
Source: Front Microbiol. 2019 Dec 3;10:2790. doi: 10.3389/fmicb.2019.02790 (PMC6901432; doi:10.3389/fmicb.2019.02790)
Supplement: Supplementary file 3 [file Data_Sheet_3.PDF]

**Suppl. Table S2**. Dose-dependent effects of omeprazole on the acyclovir concentrations that reduce HSV-1- and HSV-2-induced cytopathogenic effect (CPE) formation by 50% ($IC_{50}$) in HaCat cells. The investigated drug concentrations did not affect cell viability, neither alone, nor in combination. Omeprazole alone did not affect CPE formation.

| **HSV-1** | |
|---|---|
| Omeprazole (µg/mL) | Acyclovir $IC_{50}$ (µg/mL) |
| 0 | 0.62 ± 0.06 |
| 10 | 0.19 ± 0.05 |
| 20 | 0.074 ± 0.013 |
| 40 | 0.029 ± 0.010 |
| 80 | 0.013 ± 0.006 |

| **HSV-2** | |
|---|---|
| Omeprazole (µg/mL) | Acyclovir $IC_{50}$ (µg/mL) |
| 0 | 1.42 ± 0.17 |
| 10 | 0.45 ± 0.09 |
| 20 | 0.23 ± 0.05 |
| 40 | 0.22 ± 0.06 |
| 80 | 0.11 ± 0.03 |
