# Supplementary material for: Omeprazole Increases the Efficacy of Acyclovir Against Herpes Simplex Virus Type 1 and 2
Source: Front Microbiol. 2019 Dec 3;10:2790. doi: 10.3389/fmicb.2019.02790 (PMC6901432; doi:10.3389/fmicb.2019.02790)
Supplement: Supplementary file 4 [file Data_Sheet_4.PDF]

Suppl. Table S3. Effect of omeprazole (OME) on HSV-1 and HSV-2 titres in HaCat cells alone or in combination with acyclovir (ACV).

**HSV-1**	**Virus titre ($TCID_{50}$/mL)**	**Fold reduction relative to control**
Control	$1.3 \times 10^8 \pm 7.0 \times 10^7$	
ACV 1µg/mL	$1.2 \times 10^7 \pm 3.5 \times 10^6$	10.6
OME 10µg/mL	$7.2 \times 10^7 \pm 1.4 \times 10^7$	1.8
OME 10µg/mL + ACV 1µg/mL	$4.9 \times 10^6 \pm 1.4 \times 10^6$	26.5
OME 20µg/mL	$9.9 \times 10^7 \pm 2.7 \times 10^7$	1.3
OME 20µg/mL + ACV 1µg/mL	$5.1 \times 10^5 \pm 2.6 \times 10^5$	255
OME 40µg/mL	$5.9 \times 10^7 \pm 1.9 \times 10^7$	2.2
OME 40µg/mL + ACV 1µg/mL	$1.0 \times 10^5 \pm 5.3 \times 10^4$	1,302
OME 80µg/mL	$2.0 \times 10^6 \pm 1.9 \times 10^6$	63.9
OME 80µg/mL + ACV 1µg/mL	$8.0 \times 10^2 \pm 7.2 \times 10^2$	161,371

**HSV-2**	**Virus titre ($TCID_{50}$/mL)**	**Fold reduction relative to control**
Control	$5.1 \times 10^7 \pm 9.4 \times 10^6$	
ACV 2µg/mL	$1.1 \times 10^6 \pm 3.6 \times 10^5$	48.0
OME 10µg/mL	$2.3 \times 10^7 \pm 1.7 \times 10^6$	2.3
OME 10µg/mL + ACV 2µg/mL	$3.3 \times 10^5 \pm 2.1 \times 10^5$	156
OME 20µg/mL	$1.4 \times 10^7 \pm 5.7 \times 10^6$	3.6
OME 20µg/mL + ACV 2µg/mL	$1.1 \times 10^5 \pm 7.1 \times 10^4$	465
OME 40µg/mL	$3.1 \times 10^5 \pm 2.5 \times 10^5$	167
OME 40µg/mL + ACV 2µg/mL	$3.4 \times 10^3 \pm 4.7 \times 10^3$	15,278
OME 80µg/mL	$5.6 \times 10^3 \pm 3.3 \times 10^3$	9226
OME 80µg/mL + ACV 2µg/mL	0	N/A[1]

[1] not applicable
