# Supplementary material for: Omeprazole Increases the Efficacy of Acyclovir Against Herpes Simplex Virus Type 1 and 2
Source: Front Microbiol. 2019 Dec 3;10:2790. doi: 10.3389/fmicb.2019.02790 (PMC6901432; doi:10.3389/fmicb.2019.02790)
Supplement: Supplementary file 5 [file Data_Sheet_5.PDF]

Suppl. Table S4. Dose-dependent effects of different proton pump inhibitors on the acyclovir concentrations that reduce HSV-1-induced cytopathogenic effect (CPE) formation by 50% (IC_{50}) in HaCat cells. The investigated drug concentrations did not affect cell viability, neither alone, nor in combination. The proton pump inhibitors alone did not affect CPE formation.

	Acyclovir IC_{50} (µg/mL)			
Proton pump inhibitor	Acyclovir alone	+ proton pump inhibitor (20µM)	Acyclovir alone	+ proton pump inhibitor (10µM)
Pantoprazole	0.66 ± 0.11	0.09 ± 0.01 (7.30)[1]	0.60 ± 0.08	0.19 ± 0.08 (3.18)
Rabeprazole	0.58 ± 0.08	0.17 ± 0.04 (3.35)	0.64 ± 0.06	0.36 ± 0.10 (1.79)
Lansoprazole	0.60 ± 0.03	0.20 ± 0.08 (3.07)	0.63 ± 0.11	0.25 ± 0.08 (2.54)
Dexlansoprazole	0.62 ± 0.04	0.15 ± 0.01 (4.13)	0.59 ± 0.04	0.28 ± 0.06 (2.11)

[1] fold sensitisation relative to acyclovir alone
